# Biosynthesis of SUMOylated Proteins in Bacteria Using the *Trypanosoma brucei* Enzymatic System

**DOI:** 10.1371/journal.pone.0134950

**Published:** 2015-08-10

**Authors:** Paula Ana Iribarren, María Agustina Berazategui, Juan José Cazzulo, Vanina Eder Alvarez

**Affiliations:** Instituto de Investigaciones Biotecnológicas Dr. Rodolfo A. Ugalde-Instituto Tecnológico de Chascomús (IIB-INTECH), Universidad Nacional de San Martín (UNSAM)-Consejo Nacional de Investigaciones Científicas y Técnicas (CONICET). Campus Miguelete, Av. 25 de Mayo y Francia, 1650 San Martín, Buenos Aires, Argentina; University of Texas Medical School at Houston, UNITED STATES

## Abstract

Post-translational modification with the Small Ubiquitin-like Modifier (SUMO) is conserved in eukaryotic organisms and plays important regulatory roles in proteins affecting diverse cellular processes. In *Trypanosoma brucei*, member of one of the earliest branches in eukaryotic evolution, SUMO is essential for normal cell cycle progression and is likely to be involved in the epigenetic control of genes crucial for parasite survival, such as those encoding the variant surface glycoproteins. Molecular pathways modulated by SUMO have started to be discovered by proteomic studies; however, characterization of functional consequences is limited to a reduced number of targets. Here we present a bacterial strain engineered to produce SUMOylated proteins, by transferring SUMO from *T*. *brucei* together with the enzymes essential for its activation and conjugation. Due to the lack of background in *E*. *coli*, this system is useful to express and identify SUMOylated proteins directly in cell lysates by immunoblotting, and SUMOylated targets can be eventually purified for biochemical or structural studies. We applied this strategy to describe the ability of *Tb*SUMO to form chains *in vitro* and to detect SUMOylation of a model substrate, PCNA both from *Saccharomyces cerevisiae* and from *T*. *brucei*. To further validate targets, we applied an *in vitro* deconjugation assay using the *T*. *brucei* SUMO-specific protease capable to revert the pattern of modification. This system represents a valuable tool for target validation, mutant generation and functional studies of SUMOylated proteins in trypanosomatids.

## Introduction

SUMOylation is a post-translational modification that involves the covalent conjugation of the Small Ubiquitin-like Modifier (SUMO) to a diverse number of target proteins. The functional consequences of SUMO modification are various, typically involving alterations in subcellular localization, stability, activity or protein-protein interactions of the modified substrates [1,2]. SUMOs are ~12 kDa proteins that belong to the Ubiquitin-like proteins (UbLs) family. Although SUMO shares low amino acid sequence identity with ubiquitin (Ub) (~20%), these proteins present an almost identical structural fold with exception of an unstructured N-terminal extension that is present in SUMO and absent in other UbLs and Ub itself [3].

SUMO proteins are conserved and ubiquitously expressed in eukaryotes, but absent in prokaryotes and archaea. Generally, lower eukaryotes such as yeast or invertebrates have a single SUMO gene, while in vertebrates and plants different SUMO paralogs are expressed. Some SUMO isoforms, such as SMT3 in yeast and SUMO 2/3 in vertebrates, can polymerize and form polySUMO chains *in vivo* and *in vitro* usually via Lys residues that conform a SUMOylation consensus motif and are often found at the N-terminal region [4,5].

SUMOylation occurs via a conjugation pathway analogous to ubiquitylation which involves a three step enzymatic cascade. Initially SUMO is synthesized as an inactive precursor that must be processed by specific proteases in order to expose the C terminal di-glycine motif, necessary for conjugation. The mature form of SUMO is then activated in an ATP dependent reaction by a SUMO specific E1 activating enzyme (heterodimer E1a/E1b) [6] and a thioester bond is formed between the terminal glycine residue and the catalytic cysteine residue of E1b subunit. SUMO is then transferred by transesterification to a cysteine residue of a single E2 conjugating enzyme (Ubc9). Contrary to ubiquitin-specific E2 enzymes, Ubc9 can directly interact with SUMO substrates by recognizing SUMOylation consensus motifs and catalyze the formation of an isopeptide bond between SUMO C-terminal glycine residue and the ε-amino group of an acceptor lysine in the target protein [7]. Due to this peculiarity E1 and E2 enzymes are sufficient for *in vitro* SUMO attachment [8]. Nevertheless, the conjugation process can be assisted by SUMO E3 ligases which usually promote the interaction of E2-SUMO with the substrate or act by positioning SUMO in a conformation that facilitates its transfer to the target lysine residue [9]. Thus SUMO conjugation is generally increased in the presence of E3 ligases *in vivo* and *in vitro*.

This post-translational modification can be reverted by SUMO proteases that cleave the isopeptide bond between SUMO and its substrate; some of them are at the same time responsible for SUMO maturation. Currently all the SUMO specific proteases identified are cysteine proteases that can be classified in three classes: Ulp/SENP [10] proteases, Desi proteases [11] and USPL1 protease [12]. The Ulp/SENP family is the most studied group up to date and shows the broadest substrate specificity. These enzymes possess a conserved C-terminal domain (C48) which presents the catalytic triad His-Asp-Cys. In yeast only two deSUMOylating proteases have been identified (Ulp1 and Ulp2) and both of them belong to this class. In mammals six proteases belonging to this class are found, four of them (SENP-1, SENP-2, SENP-3 y SENP-5) present similarity with Ulp1 while the other two (SENP-6 and SENP-7) are more closely related to Ulp2. All Ulp/SENP enzymes possess isopeptidase activity being able to remove SUMO from substrates although they have different substrate specificity and play different roles in the SUMOylation pathway. Ulp1 and its homologues are C-terminal hydrolases that carry out SUMO precursor processing; while Ulp2, SENP-6 and SENP-7 are the main enzymes responsible for polySUMO chain edition [13].

Trypanosomatids are parasitic protists that cause serious neglected diseases to man, affecting a high number of people worldwide; *Trypanosoma cruzi* is the causative agent of Chagas disease in South America while *Trypanosoma brucei* is the ethiological agent of African sleeping sickness. Like in other invertebrates, a single SUMO gene has been identified in these parasites.

*T*. *brucei* SUMO (*Tb*SUMO) shares 33% and 37% similarity with yeast SUMO (Smt3) and human SUMO-1 [14]. Like this latter isoform *Tb*SUMO does not contain internal SUMOylation consensus sites and it is still unknown whether it is able to form SUMO chains. The 3D structure of *Tb*SUMO has been solved by NMR, showing that *Tb*SUMO structure is highly similar to all other SUMOs and it is able to interact with human Ubc9 in a unique way through a Thr residue (T30) in the N-terminal extension [14]. By performing RNAi experiments it could be determined that *Tb*SUMO is an essential protein for both replicative life-cycle stages of the parasite, procyclic (PCF) and bloodstream (BSF) forms. In PCF parasites SUMO absence inhibits mitosis, arresting cells in G2/M phase and affects chromosome segregation [15], while in SUMO deficient BSF parasites inhibition of cytokinesis could be observed [16]. Notably, in contrast to other eukaryotic organisms Trypanosomes contain a limited number of general transcription factors what suggests lack of regulation at transcriptional level. In this way post-transcriptional and post-translational modifications, including SUMOylation, may play fundamental roles in regulation of gene expression and coordination of diverse cellular processes necessary for parasite differentiation and survival. In *T*. *brucei* SUMO has been associated to regulation of antigenic variation of the parasite surface glycoprotein coat, a process where the major surface antigenic protein is replaced by a different variant with certain frequency, protecting the parasite from the host immune response. SUMO was found to be enriched in a particular region of the nucleus of BSF parasites together with the E3 ligase Siz1 and the RNApolI, specifically at the chromatin region that is actively transcribing the variant surface glycoprotein suggesting that SUMOylated unknown factors are involved in VSG mono allelic active expression [17].

In spite of its importance, only four trypanosomatid proteins have been experimentally demonstrated to be SUMOylated so far: *Tc*MCA3 [18] and PAR2 [19] in *T*. *cruzi*, and Aurora kinase B [20] and RPA1 in *T*. *brucei* [17]. Usually, identification of proteins modified by SUMO is performed by high-throughput proteome analysis by mass spectrometry, but *in vitro* assays are often necessary for the study and validation of the actual SUMOylation of specific substrates. For this reason, the development of a specific SUMOylation system is a fundamental tool for the study of this post-translational modification. In the present work, we have succeeded in confirming the activity and conjugation ability of the enzymes *Tb*E1 and *Tb*E2, and in developing a specific system for the validation of SUMOylated proteins in *T*. *brucei*, by combining the reconstitution of the SUMOylation machinery of this parasite in *Escherichia coli* with *in vitro* deconjugation assays.

## Results

### Reconstitution of the *T*. *brucei* SUMOylation machinery in *E*. *coli*

**Fig 1:**
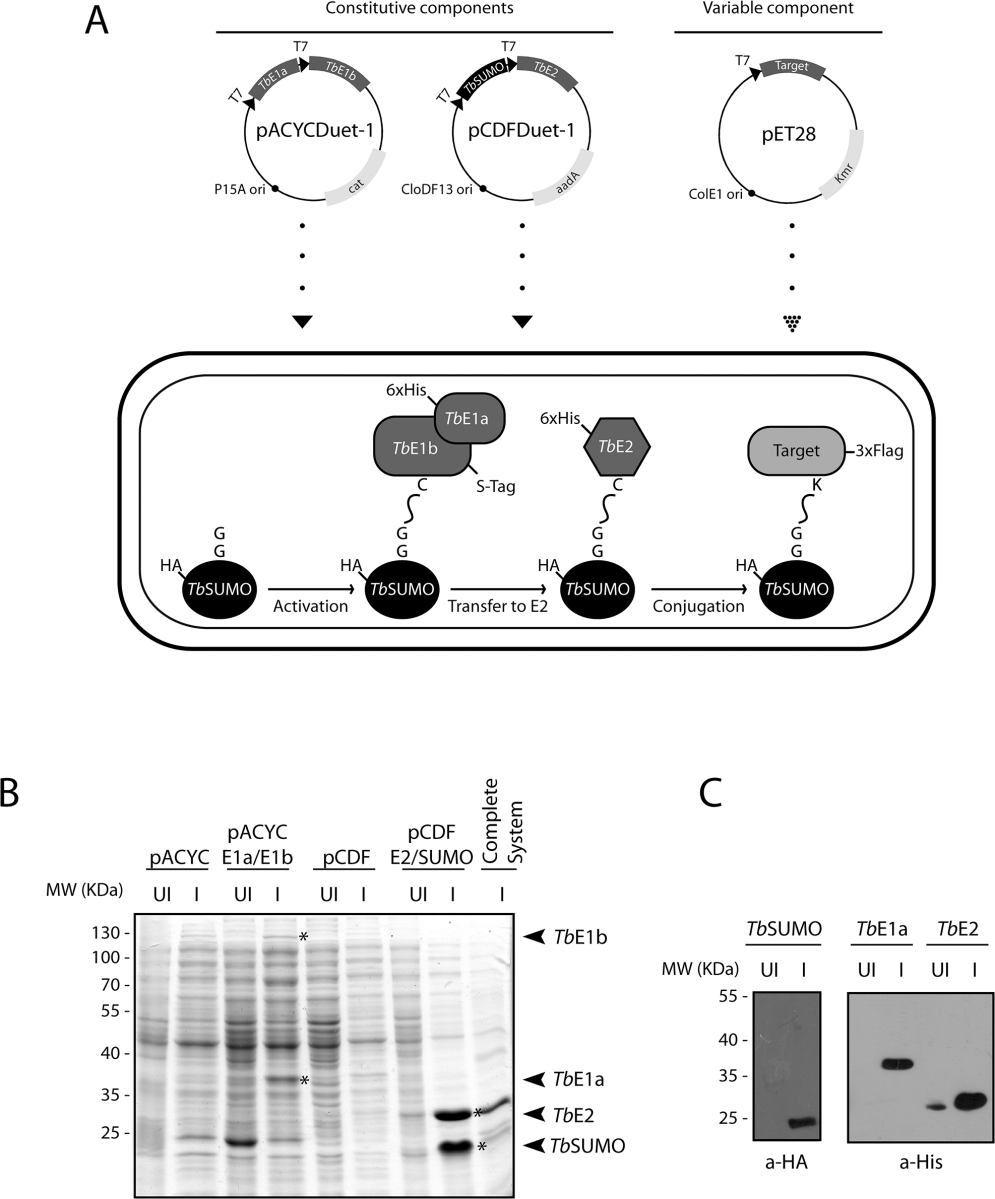
Reconstitution of the *T*. *brucei* SUMOylation system in *E*. *coli*. **(A)** Schematic representation of the plasmids used to express the multiple components of the *T*. *brucei* SUMOylation system in bacteria. pACYCDuet-1 carrying the P15A replicon and chloramphenicol resistance gene (*cat*) was designed to direct the co-expression of *T*. *brucei* activating enzyme subunits a and b (*Tb*E1a and *Tb*E1b) as fusions to an N-terminal His-tag or a C-terminal S-tag, respectively. pCDFDuet-1 carries the CloDF13 replicon and the streptomycin/spectinomycin resistance gene (*aadA*) and drives the co-expression of mature SUMO (*Tb*SUMO) and its conjugating enzyme (*Tb*E2) both tagged at the N-terminus either with an HA epitope or a His tag, respectively. Finally, pET28 carries the ColE1 origin of replication and the kanamycin resistance gene (*Kmr*) and is used for individual expression of SUMOylation target proteins. **(B)** Analysis of recombinant protein expression by Coomassie Blue staining of SDS-PAGE gels. Equal amount of protein (30 μg) was loaded in each lane. Samples correspond to the soluble fraction of *E*. *coli* BL21 (DE3) host cells transformed with the empty vector pACYCDuet-1 (lanes 1 and 2),pACYCDuet-1-*Tb*E1a-*Tb*E1b (lane 3 and 4), pCDFDuet-1 (lane 5 and 6), pCDFDuet-1-*Tb*SUMO-*Tb*E2 (lane 7 and 8), or with the complete SUMOylation system (lane 9) and induced (I) or not (UI) for protein expression during 5 hr at 37°C using 1mM IPTG. The predicted molecular masses of the recombinant proteins (including tags) are: 14 kDa for *Tb*SUMO, 28 kDa for *Tb*E2, 40 kDa for *Tb*E1a and 97 kDa for *Tb*E1b. Recombinant proteins are marked with an asterisk in the figure and labeled with an arrowhead at the right of the gel. **(C)** Immunoblot detection of the recombinant proteins was performed on the same samples using anti-HA antibodies for *Tb*SUMO and anti-His antibodies for *Tb*E2 and *Tb*E1a.

To promote the recombinant expression of *T*. *brucei* SUMOylated proteins in bacteria, we decided to transfer the complete set of enzymes essential for this post translational modification in this organism to *E*. *coli*. Fig 1A illustrates the triple vector system, with compatible origins of replication and independent antibiotic selection, designed to inducibly co-express both subunits of the SUMO activating enzyme (*Tb*E1a and *Tb*E1b) from the Duet vector pACYCDuet-1, together with processed SUMO (*Tb*SUMO) and its conjugating enzyme (*Tb*E2) from other Duet vector pCDFDuet-1 and lastly, the target of SUMOylation from vector pET28.

We started evaluating the correct expression and solubility of the individual components in bacterial lysates by SDS-PAGE followed by Coomassie Blue staining. As shown in Fig 1B, we observed that *Tb*SUMO and the conjugating enzyme were highly expressed in *E*. *coli*, while the activating enzyme subunits, and in particular *Tb*E1b, were produced at significantly lower levels [21]. Recombinant expression was further confirmed for the proteins expressed as N-terminal fusions to either His tag (*Tb*E1a and *Tb*E2) or HA epitope (*Tb*SUMO) by Western blot analysis using commercially available antibodies (Fig 1C). All recombinant proteins migrated as single bands at the expected molecular weight with the exception of SUMO, for which an anomalous electrophoretic behavior has already been described [22].

**Fig 2:**
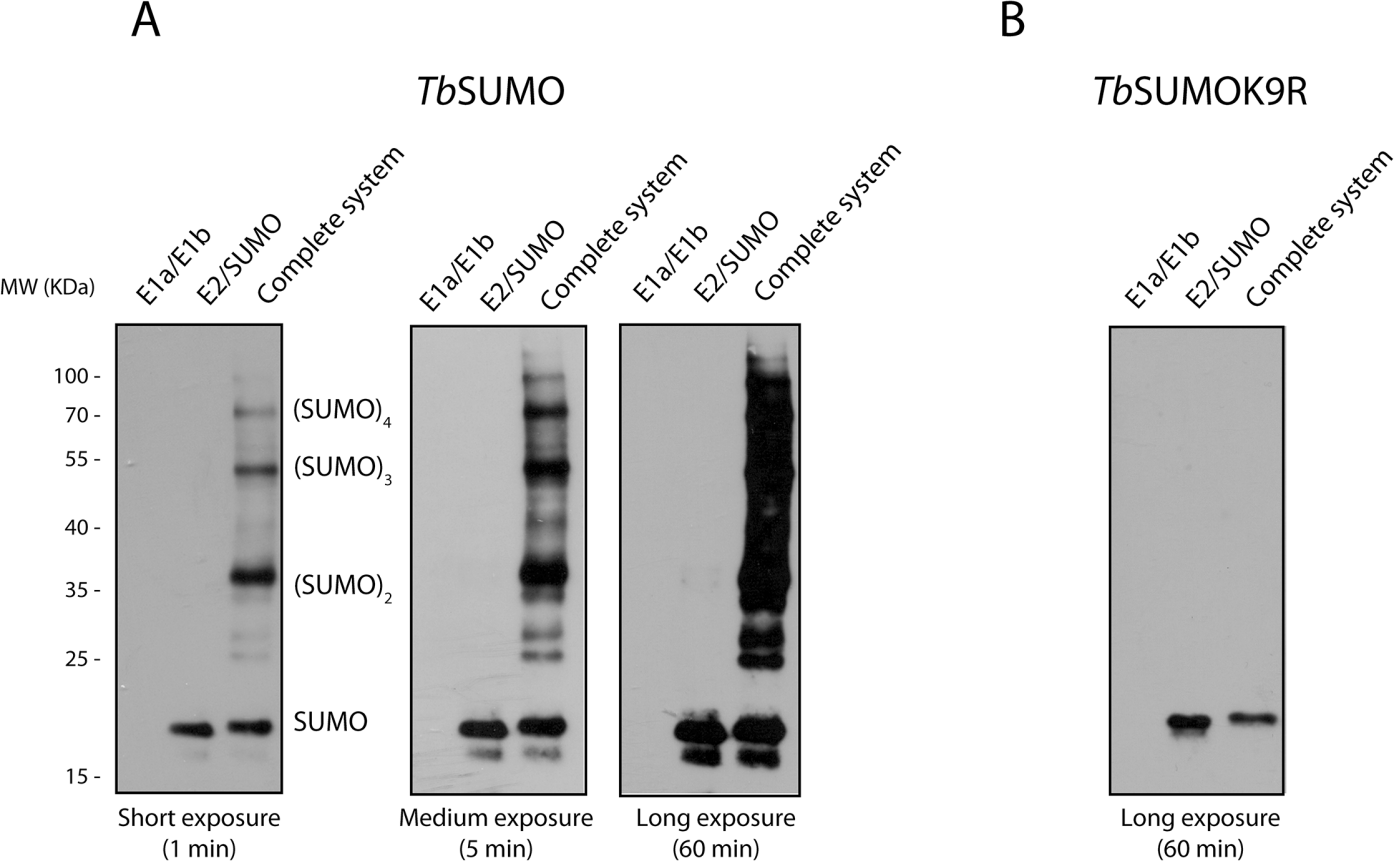
*Tb*SUMO chain formation. **(A)** Anti-HA Western blot analysis of soluble cell extracts from induced cultures of *E*. *coli* transformed with only one plasmid pCDFDuet-1-*Tb*SUMO-*Tb*E2; pACYCDuet-1-*Tb*E1a-*Tb*E1b or both pCDFDuet-1-*Tb*SUMO-*Tb*E2 and pACYCDuet-1-*Tb*E1a-*Tb*E1b. Different exposure times were used to evidence the SUMO ladder which was observed at the shorter times while a more complex pattern was obtained with longer periods of exposure. *Tb*SUMO monomer, dimers, trimers and multimers are indicated. **(B)** Western blot analysis of SUMO pattern performed on soluble cell extracts from an incomplete (lanes 1 and 2) or a complete bacterial SUMOylation system (lane 3) using a Lys deficient version of SUMO (*Tb*SUMO K9R). Note the complete absence of SUMO conjugates implying the absence or artificial SUMOylation of bacterial proteins.

We next assessed the performance of the system in the absence of a SUMO target. Western blot analysis using anti-HA (Fig 2A) or anti-*Tb*SUMO (S1 Fig) antibodies of the *Tb*SUMO pattern of cells transformed just with plasmid pCDFDuet-1-*Tb*SUMO-*Tb*E2 showed that *Tb*SUMO was present as a single band; however, when the two plasmids (pCDFDuet-1-*Tb*SUMO-*Tb*E2 and pACYCDuet-1-*Tb*E1a-*Tb*E1b) were used to transform *E*. *coli*, additional slowly migrating bands likely corresponding to *Tb*SUMO multimers were detected. It has been previously reported that SUMO from *T*. *cruzi* possess two internal SUMOylation sites that can be modified by human SUMO-1 in *E*. *coli*, one of them possibly located in a typical consensus motif while the other occurring in a yet non-identified Lys residue [19]. To discriminate if SUMO chain formation could also take place in *T*. *brucei*, we replaced the wild-type *Tb*SUMO in our bacterial assay with a Lys deficient version of the protein (in which all Lys residues were mutated to Arg and therefore cannot be substrate for SUMOylation) and performed a similar Western blot analysis. As shown in Fig 2B, the majority of the higher molecular weight proteins were now undetectable confirming that they indeed correspond to polySUMO chains.

### *In vivo* heterologous SUMOylation of a model substrate using *T*. *brucei* system

**Fig 3:**
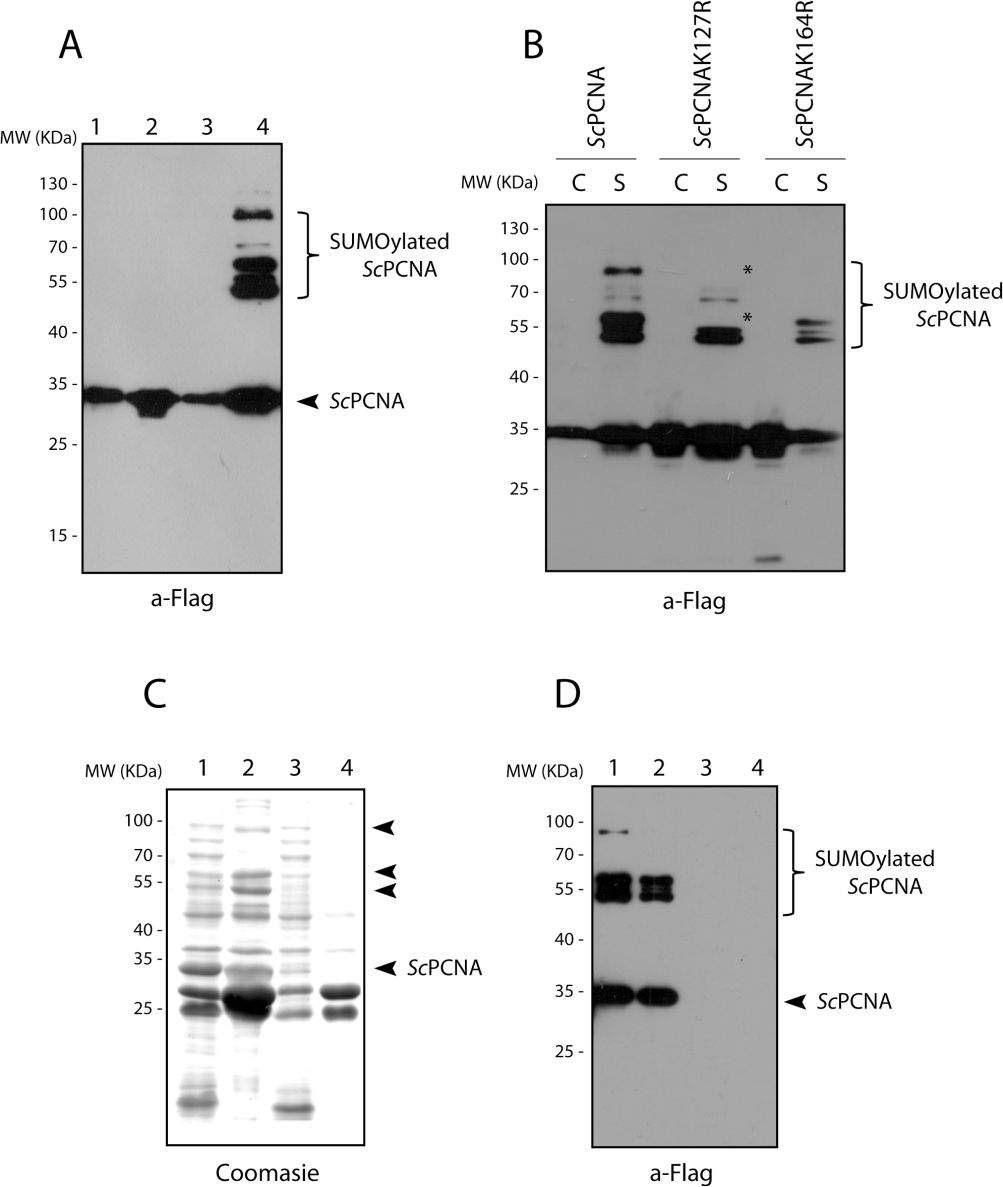
In-bacteria SUMOylation of *Sc*PCNA. **(A)** Anti-Flag Western blot analysis of *Sc*PCNA performed on soluble cell extracts from induced cultures of *E*. *coli* transformed with pET28-*Sc*PCNA-3xFlag alone (lane 1) or in the background of an incomplete (lane 2, pACYCDuet-1-*Tb*E1a-*Tb*E1b; lane 3, pCDFDuet-1-*Tb*SUMO-*Tb*E2) or a complete (lane 4, pCDFDuet-1-*Tb*SUMO-*Tb*E2 plus pACYCDuet-1-*Tb*E1a-*Tb*E1b) SUMOylation system. **(B)** Mutational analysis of *Sc*PCNA was performed in a background of *E*. *coli* Bl21 (DE3) cells (C) or in a complete SUMOylation system (S). The band corresponding to SUMO conjugated to K127 and the higher molecular weight band corresponding to poliSUMOylated PCNA at K127 or K164 are marked with asterisks. (**C**) Bacterial lysate overexpressing SUMOylated *Sc*PCNA or the complete SUMOylation system as a control, were subjected to Ni^+2^ affinity chromatography. The inputs (lane 1 and 3) and the eluates (lane 2 and 4) were analyzed by Coomassie staining or immunoblotting using monoclonal anti-Flag antibodies.

We validated the functionality of the "*in bacteria*" *T*. *brucei* SUMOylation system introducing the third vector which directs the expression of a well-established target of SUMO, the proliferating cell nuclear antigen (PCNA) from *Saccharomyces cerevisiae* (see below) and from *T*. *brucei* (S3 Fig), fused to a triflag epitope at the C-terminus. Protein expression was induced with 1 mM IPTG at 37°C for 5 hr, and cell lysates were analyzed by Western blot using anti-Flag antibodies. *Sc*PCNA can be obtained with high yield and appears as a single band with the expected size when expressed alone in *E*. *coli* (Fig 3A, lane 1). However, when co-expressed with *Tb*SUMO, *Tb*E1a/*Tb*E1b and *Tb*E2 enzymes, two additional slower-migrating bands can be detected (Fig 3A, lane 4). These bands were not visible when *Sc*PCNA was co-expressed with the partially reconstituted system, which were used as negative controls (Fig 3A, lane 2 and 3). Additional controls are shown in S2 Fig.

The SUMOylation pattern of *Sc*PCNA has been extensively studied [23]. Two lysine residues were unambiguously identified as SUMO targets (K127 and K164), while some other/s lysine residue/s seem also to be modified by SUMO but were not identified by mutational analysis to date. To compare our SUMOylation pattern with these observations we independently mutated the K127 or K164 to arginine, and analyzed the changes in the *Sc*PCNA pattern by Western blot analysis using anti-Flag antibodies. As shown in Fig 3B, the wild type and the mutant versions were expressed correctly when transformed individually in BL21 (DE3) cells. When the K127R mutant was transformed together with the complete SUMOylation system we observed that the upper band of the triplet around 55 kDa disappeared suggesting that this band can be attributed to the modification of this residue. On the other hand, the triplet was again visible when analyzing the K164R mutant transformed together with the complete SUMOylation system, suggesting that this residue is not responsible for any of these bands. However, a clearly decrease in the intensity of the higher molecular weight bands (~100 kDa) was observed for these mutants likely suggesting that this band could correspond to multi or poli-SUMOylation of PCNA both in K127 and in K164.

To demonstrate that *Sc*PCNA is actually being SUMOylated we repeated the experiment using a different *Tb*SUMO construct. We employed a His-HA-tagged *Tb*SUMO variant that allows purification of SUMOylated PCNA using Ni^2+^ affinity chromatography and immunoblot detection of the SUMOylated protein using anti-Flag antibodies. As shown in Fig 3C and 3D the same doublet is reactive with both anti-Flag and anti-HA antibodies confirming that they certainly correspond to SUMO covalently linked to PCNA.

### Production of recombinant *Tb*SENP: evaluation of peptidase and isopeptidase activities

**Fig 4:**
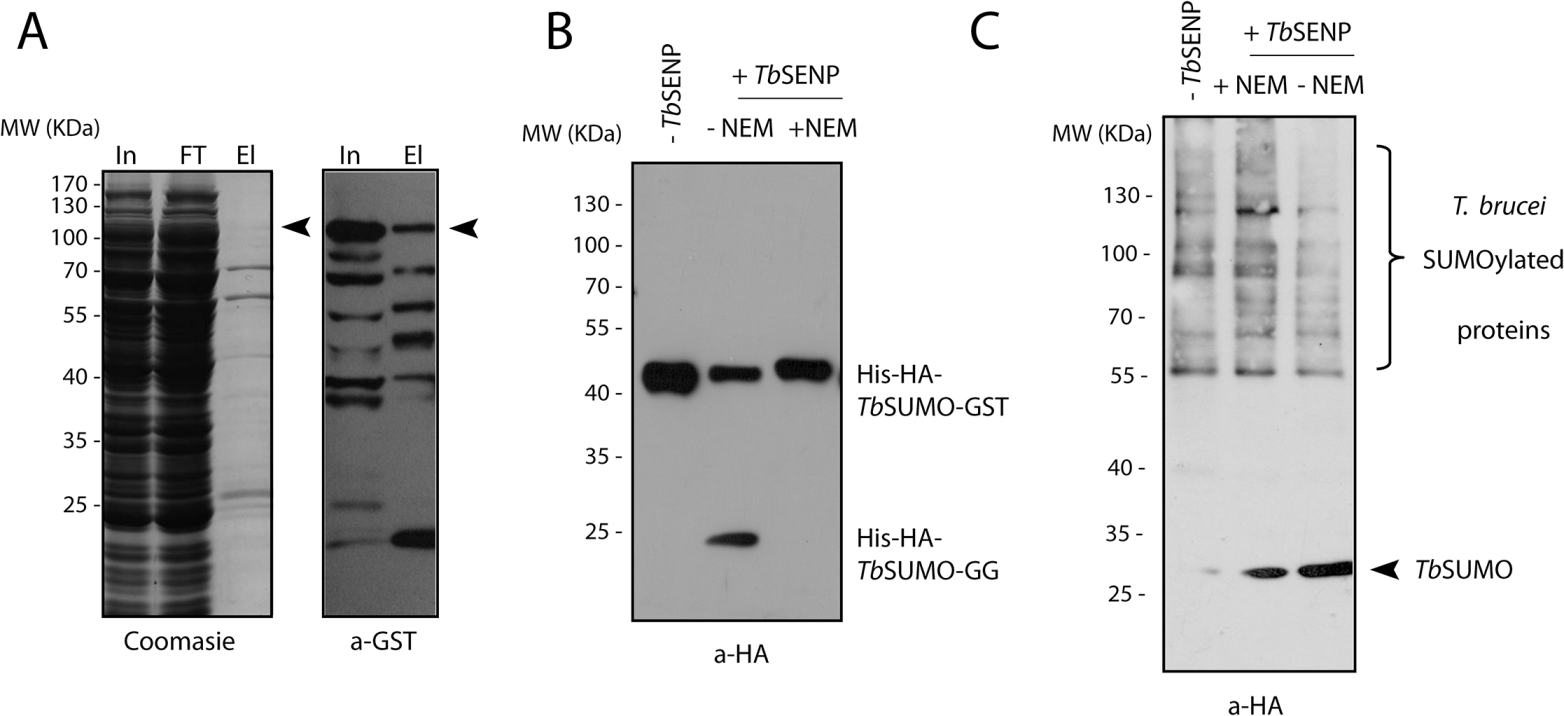
*Tb*SENP peptidase and isopeptidase activity. **(A)** SDS-PAGE followed by Coomassie Blue staining (left panel) or Western blot analysis using anti-GST antibodies of *Tb*SENP purification (right panel). In: input, cell-free extract from bacteria overexpressing *Tb*SENP, FT: Flow through, fraction not retained by the resin and El: eluate, sample retained and eluted from the resin. The full length protein is marked with an arrowhead. Faster migrating bands likely correspond to *Tb*SENP-GST degradation products. **(B)** SUMO precursor cleavage by *Tb*SENP was evaluated *in vitro* using a *Tb*SUMO precursor produced in *E*. *coli* tagged at the N-terminus with His-HA and fused at the C-terminus to the GST protein. After purification on glutathione-agarose resin 7.5 μg of His-HA-*Tb*SUMO-GST protein was mixed with 0.75 μg of purified recombinant *Tb*SENP (produced as described in Materials and Methods) in 30 μl of TBS containing 1 mM DTT in the absence (lane 2) or presence (lane 3) of the general cysteine peptidase inhibitor N-ethylmaleimide 20 mM final concentration (NEM) and incubated at 37°C for 1 hr. Samples were analyzed by Western blot using anti-HA monoclonal antibodies. The substrate without the addition of the protease was run as a control (lane 1). **(C)** Broad-specificity SUMO deconjugation ability of *Tb*SENP was analyzed on purified HA-tagged *Tb*SUMO conjugates from parasites (See Materials and Methods). Isopeptidase activity was evaluated in reaction mixtures containing 3 μg of *Tb*SUMO conjugates and 0.75 μg of purified *Tb*SENP in 30 μl of TBS containing 1 mM DTT in the presence (lane 2) or in the absence (lane 3) of 20 mM NEM incubated at 37°C for 1 hr. Samples were analyzed by Western blot using anti-HA monoclonal antibodies. The substrates without the addition of the protease was run as a control (lane 1).

To add confidence to our bacterial SUMOylation assay, we decided to specifically revert this modification using a recombinant SUMO specific deconjugating enzyme from *T*. *brucei*. To develop this tool we cloned, expressed and purified *Tb*SENP in *E*. *coli* C-terminally fused to glutathione S-transferase (GST) (Fig 4A).

We first tested the ability of this peptidase to cleave a double-tagged *Tb*SUMO precursor containing an N-terminal HA epitope and a C-terminal GST. HA-*Tb*SUMO-GST was incubated with *Tb*SENP at 37°C for 1 hr and the reaction products were analyzed by Western blot using anti-HA antibodies (Fig 4B) and anti-GST antibodies (S4 Fig). Fig 4B shows that *Tb*SENP cleaves the *Tb*SUMO precursor resulting in a cleavage product of about 25 kDa, compatible with proteolytic processing taking place after the diGly motif. The cleavage was inhibited in the presence of a general inhibitor of cysteine peptidases, iodoacetamide (NEM).

We next assessed the ability of *Tb*SENP to deconjugate *Tb*SUMO from a broad spectrum of substrates. We used a transgenic cell line of procyclic parasites in which the endogenous *Tb*SUMO locus was replaced with a His-HA tagged gene allowing efficient purification of *Tb*SUMO conjugates by nickel affinity chromatography. We mixed purified *Tb*SUMO targets with *Tb*SENP and incubated the reaction for 1 hr at 37°C. Western blot analysis of the samples using anti-HA antibodies showed an increase of free *Tb*SUMO protein intensity in parallel with a marked reduction of *Tb*SUMO conjugates that was not observed when NEM was added to the reaction mixtures (Fig 4C).

Altogether these results demonstrate that *Tb*SENP is an active cysteine peptidase that, as described for its yeast and mammalian orthologues [10,24,25,26,27], is able both to process *Tb*SUMO precursor (peptidase activity) and to deconjugate *Tb*SUMO from its various targets (isopeptidase activity).

### Target validation using bacterial SUMOylation assay coupled to *in vitro* deSUMOylation using *T*. *brucei* components

**Fig 5:**
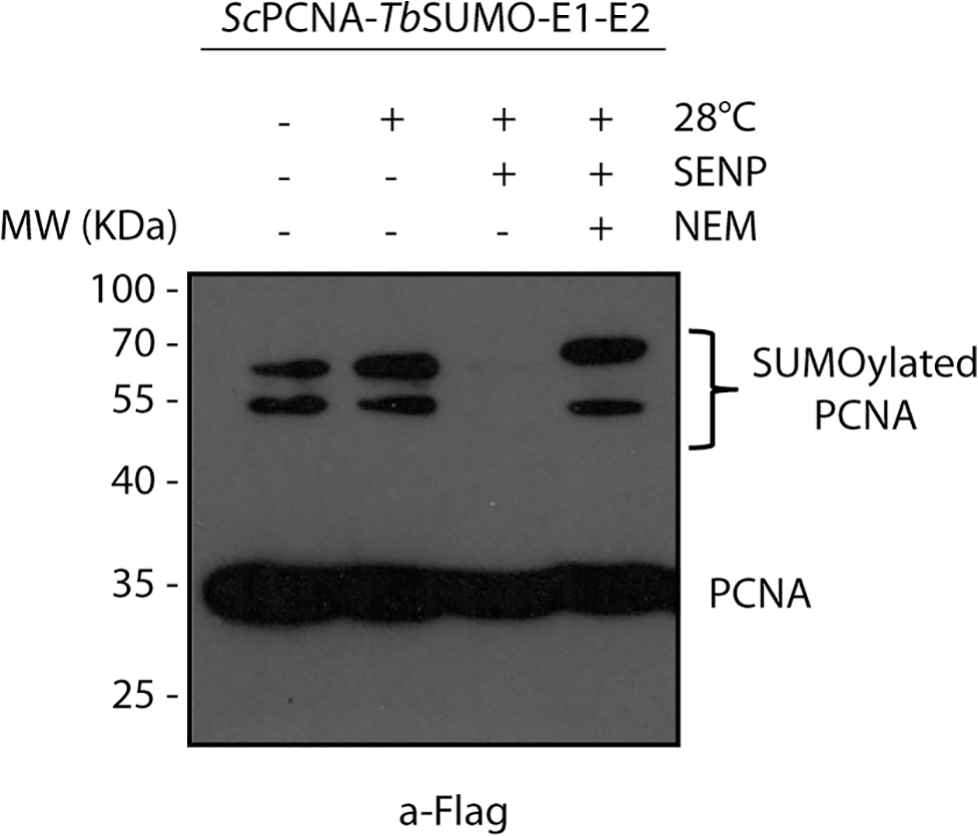
*In vitro* deconjugation of SUMOylated *Sc*PCNA. Cell lysates of *E*. *coli* heterologously expressing *Sc*PCNA and the complete *T*. *brucei* SUMOylation system (lane 1) were incubated at 28°C in the absence (lane 2) or presence of recombinant *Tb*SENP (lane 3) as described in Material and Methods. Deconjugation ability of *Tb*SENP was specifically inhibited by the addition of 20 mM NEM (lane 4). Reaction mixtures were analyzed by Western blot using anti-Flag monoclonal antibodies.

We finally demonstrated the function of *Tb*SENP in confirming a substrate of SUMOylation. As shown in Fig 5, the two additional slowly migrating bands observed when *Sc*PCNA was co-expressed with the *T*. *brucei* SUMOylation system in bacteria completely disappeared upon treatment of cell lysates with *Tb*SENP, confirming that they indeed correspond to SUMOylated *Sc*PCNA proteins. Thus, combining bacterial SUMOylation assays with *in vitro* deconjugation reactions represents a suitable strategy to validate SUMO targets.

## Discussion

We have succeeded in establishing a recombinant system to produce SUMOylated proteins in bacteria by transferring the minimal set of enzymes needed for this modification in *T*. *brucei* to *E*. *coli*. We co-expressed both activating enzyme subunits together with the conjugating enzyme and processed *Tb*SUMO (already exposing the diGly motif) using two different Duet vectors, while a compatible pET28 vector was used to express the target protein of interest. The addition of an appropriate tag in the target allows the detection of the SUMOylated form by Western blot analysis directly on cell lysates without any enrichment step. Moreover, the addition of another tag on *Tb*SUMO makes feasible a tandem affinity purification of the modified target. However, tags should be chosen carefully when biochemical and/or structural studies of the SUMOylated proteins are planned to be undertaken [28,29].

This bacterial SUMOylation system is useful not only to validate the SUMOylation sites found in proteomic experiments, but also to generate and test non-SUMOylable mutants, a task that can be sometimes laborious due to the appearance of cryptic SUMOylation sites upon mutation of the canonical ones [30,31,32]. We generated and evaluated the performance of a His-HA-*Tb*SUMO protein with a mutation of the Thr residue at position 106 (prior to the diGly motif) to Lys (S5 Fig). This *Tb*SUMO version is useful to map acceptor Lys in substrates by MS/MS locating the remnant diGly after a subsequent trypsin or Lys-C digestion.

This *in bacteria* SUMOylation reaction is simple, fast and economically convenient when compared to the *in vitro* reactions using commercially available or "in-house purified" human or yeast recombinant enzymes [21,28,29,33,34]. Furthermore, this tool has been specifically designed for trypanosomatid proteins and can potentially avoid subtle differences in specificity (i.e., the Lys residue that is being modified or the ability to form chains) when using a source of enzyme from a different organism. While this work was being revised, a paper by Ye *et al*. reported the development of an *in vitro* system for *T*. *brucei* [35].

In this assay we chose *Sc*PCNA as a model target, a well-known SUMOylation substrate that has been employed also as heterologous control for other *in vitro* SUMOylation systems in other organisms [36]. PCNA is a processivity factor of DNA polymerase and its SUMOylation has been associated *in vivo* with genome maintenance [37]. The modification pattern observed using this *in vivo* reconstituted SUMOylation system resembles the one obtained with the *in vitro* reconstituted SUMOylation machinery of *Saccharomyces cerevisiae* [23].

With the reconstituted system, we confirmed the functionality of the *Tb*E1 and *Tb*E2 proteins that were annotated as putative enzymes [18,38]. We further studied another vital component of the SUMOylation/deSUMOylation regulatory cycle, which is the SUMO specific peptidase *Tb*SENP. We cloned, expressed and purified the recombinant enzyme and showed that this protein: 1) possesses the ability to process *Tb*SUMO precursor making it available for activation and conjugation reactions and, 2) can revert *Tb*SUMO modification by cleaving off *Tb*SUMO from its target protein through its isopeptidase activity, an ability that can be exploited to confirm protein SUMOylation by disappearence of high molecular weight adducts after treatment with the enzyme.

Finally, we demonstrated that *Tb*SUMO forms multimers in our bacterial SUMOylation assay, an observation already suggested by the *in vitro* reactions of its *T*. *cruzi* orthologue [19]. However, *Tb*SUMO chain formation seems not to be an essential feature *in vivo* since it was possible to replace the endogenous gene with a Lys deficient version [39], similar to the situation described for *S*. *cerevisiae* [40].

## Materials and Methods

### Trypanosome culture

Parasites employed were procyclic form (PCF) *T*. *brucei brucei* Lister 427 [41] andHis-HA-*Tb*SUMO, a Lister 427 cell line with both SUMO alleles replaced by a His-HA-*Tb*SUMO variant [39].

PCF cell lines were maintained axenically at 28°C in SDM-79 medium [42] supplemented with 10% (vol/vol) heat-inactivated fetal calf serum (Natocor, Córdoba, Argentina) and 7.5 mg/l hemin.

### Genomic DNA purification and Polymerase Chain Reaction

*T*. *brucei brucei* Lister 427 PCF collected by centrifugation were washed twice with PBS (0.1 M sodium phosphate, 0.15 M NaCl, pH 7.4) and genomic DNA was isolated using DNAzol Reagent as described by the manufacturer (Life Technologies, Carlsbad, CA, USA).

Polymerase Chain Reaction (PCR) was performed at a final volume of 50 μl containing genomic (~300 ng) or plasmid DNA (~50 ng), 30 pmoles of the specific primers (Macrogen, Seoul, Korea), 2.5 mM of each deoxynucleotide triphosphate (dATP, dCTP, dGTP, dTTP) (New England Biolabs), 1.5 mM MgCl_2_ (Life Technologies), 0.2 UI *Taq* DNA polymerase (Life Technologies) and reaction buffer as indicated by the manufacturer (Life Technologies).

For the amplification reaction a thermocycler (Mycycler, Bio-Rad, Hercules, CA, USA) was employed: an initial denaturation step at 94°C for 10 min was followed by 35 cycles of a) denaturation at 94°C 1 min, b) hybridization at 55°C 1 min, c) elongation at 72°C -1 min per kb of DNA amplified; the final elongation step was performed at 72°C for 10 min.

### Plasmid constructions

The open reading frames (ORFs) of *Tb*SUMO (Tb927.5.3210), *Tb*E2 (Tb927.2.2460), *Tb*E1a (Tb427tmp.02.5410), *Tb*E1b (Tb927.5.3430) and *Tb*PCNA (Tb927.9.5190) were amplified by PCR from *T*.*brucei* genomic DNA using the following primers: HA-*Tb*SUMO sense CATATGTACCCATACGATGTTCCAGATTACGCTATGGACGAACCCACTCATAAC, *Tb*SUMO antisense CTCGAGTCACCCGCCTGTCTGCTCCACC, *Tb*E2 sense GGATCCGATGTCCGGGCTATCTTTAGC, *Tb*E2 antisense GCGGCCGCTTATACCCGCTTCCGGTG, *Tb*E1a sense GAATTCGATGAATGCGGACGAAAAAACG, *Tb*E1a anti sense AAGCTTCTACGGGTTGCGCAGGTGCC, *Tb*E1b sense CATATGCACGTTAATGTCGGACATATTGTC, *Tb*E1b antisense CTCGAGATCAATTTCTACAACCTCGTCACTATC, *Tb*PCNA sense CCATGGCCCTTGAGGCTCAGGTTCTGCAC and TbPCNA antisense CCATGGACTCGGCGTCGTCCACCTTTG.

To generate HisHA-*Tb*SUMO variants we used plasmid constructions with the complete ORF of HisHA-*Tb*SUMO or HisHA-*Tb*SUMO ORF with all Lys residues replaced by Arg (GenScript, Piscataway, NJ, USA) as template for PCR amplification using the following primers: HisHA-*Tb*SUMO sense CATATGGACGAACACCACCAC, *Tb*SUMO antisense CTCGAGTCACCCGCCTGTCTGCTC (HisHA-*Tb*SUMO variant), HisHA-*Tb*SUMO sense CATATGGACGAACACCACCAC, *Tb*SUMOK9R antisense CTCGAGTCACCCGCCCGGCTGCTC (HisHA-*Tb*SUMOK9R variant), HisHA-*Tb*SUMO sense CATATGGACGAACACCACCAC, *Tb*SUMO^T106K^ antisense CTCGAGTCACCCGCCCTTCTGCTC (HisHA-*Tb*SUMO^T106K^ variant).

Amplification products were first cloned into pGEM-T Easy vector (Promega, Madison, WI, USA). To generate the construct to express *Tb*SUMO/*Tb*SUMO variants and *Tb*E2 (pCDFDuet-1-*Tb*SUMO-*Tb*E2), the *Nde*I/ *Xho*I fragment of *Tb*SUMO was cloned into the multicloning site 2 (MCS2) of the expression vector pCDFDuet-1 (Novagen, Palo Alto, CA, USA). Subsequently, the *Bam*HI/ *Not*I fragment of *Tb*E2 was cloned into multicloning site 1 (MCS1) of the vector. To generate the construct to express *Tb*E1a and *Tb*E1b (pACYCDuet-1-*Tb*E1a-*Tb*E1b), the *Nde*I/ *Xho*I fragment of *Tb*E1b was cloned into the MCS2 of the expression vector pACYCDuet-1 (Novagen). Subsequently, the *Eco*RI/ *Hind*III fragment of *Tb*E1a was cloned into the MCS1 of the vector.

To construct the plasmid for substrate expression, the ORF of *Saccharomyces cerevisiae* proliferating cell nuclear antigen (*Sc*PCNA) (P15873.1) was amplified by PCR from *S*.*cerevisiae* genomic DNA using as sense primer CCATGGTAGAAGCAAAATTTG and as antisense primer CCATGGATTCTTCGTCATTA. *Sc*PCNA ORF was first cloned into pGEM-T Easy vector (Promega) and then into pBAD-3xFlag vector through *Bam*HI/ *Hind*III restriction sites. Then, the *Nco*I digested fragment was finally cloned into the expression vector pET28a (Novagen) to express C-terminal 3xFlag *Sc*PCNA (pET28a-*Sc*PCNA). To generate *Sc*PCNA SUMOylation mutants (*Sc*PCNAK127R and *Sc*PCNAK164R) the following primers were used to amplify by PCR different fragments from *Sc*PCNA ORF in pGEM-T Easy vector: *Sc*PCNAK127R sense GATGCTGATTTCTTAAAGATTGAAGAATTACA, *Sc*PCNAK127R antisense TGTAATTCTTCAATGCGTAAGAAATCAGCATC, *Sc*PCNAK164R sense AATATCATGATCACCAAAGAAACAATAAAGTTTG and *Sc*PCNAK164R antisense CAAACTTTATTGTTTCACGGGTGATCATGATATT. The final amplification products were cloned into pGEM-T Easy vector (Promega) and sequenced to confirm the presence of the mutated codon (Macrogen). These constructions were then cloned into the expression vector pET28a-3xFlag to express C-terminal 3xFlag fusion proteins through *Nco*I restriction sites. The same strategy was used to express *Tb*PCNA-3xFlag (pET28a-*Tb*PCNA).

For *Tb*SENP (Tb927.9.2220) expression the complete ORF was amplified by PCR using CCCGGGTATGGCAGATATCCTTTTAAATGCCG as sense primer and ACTAGTCGCCTTCGTGTATAAGGCCAGT as antisense primer and cloned into pGEM-T Easy vector (Promega) and then into pBAD-GST vector through *Sma*I/ *Spe*I restriction sites.

For His-HA-*Tb*SUMO-GST expression the complete ORF of *Tb*SUMO was amplified by PCR using CCATGGACGAACACCACCAC as sense primer, ACTAGTCGCCATGCACCAAAGACACCCGCCTGTCTGCTCC as antisense primer and a plasmid construction with the complete ORF of HisHA-*Tb*SUMO as template. The amplified fragment was cloned into pGEM-T Easy vector (Promega) and digested with *Nco*I and *Spe*I (New England Biolabs, Ipswich, MA, USA) for cloning into pBAD-GST vector.

The identity of the constructions described was confirmed by sequencing (Macrogen).

### *In vivo* reconstituted SUMOylation

*Escherichia coli* BL21 (DE3) cells were transformed with pCDFDuet-1-*Tb*SUMO/*Tb*SUMO variants-*Tb*E2 and used for the preparation of Calcium Chloride competent cells. These cells were transformed with pACYCDuet-1-*Tb*E1a-*Tb*E1b and competent bacteria were made again to transform with pET28a-*Sc*PCNA, pET28a-*Sc*PCNAK127R, pET28a-*Sc*PCNAK164R or pET28a-*Tb*PCNA. To assess the SUMOylation reaction cells containing the three plasmids mentioned (or a subset of them for control experiments) were cultured in Luria–Bertani (LB) medium at 37°C to an OD_600nm_ of 0.6, and then induced with 1 mM isopropyl β-D-1-thiogalactopyranoside (IPTG) for 5 hr at 37°C with vigorous shaking (250 rpm). Cells were harvested by centrifugation and resuspended in lysis buffer (150 mM NaCl, 50 mM Tris HCl, 0.4 mg/ml lysozyme, 0.1% Triton X-100, 10 mM ethylene diamine tetraacetic acid (EDTA), 1 mM phenylmethylsulfonyl fluoride (PMSF)—pH 7.6) and sonicated when necessary. Samples were then centrifuged for 30 min at 23000 x *g* and 70 μl of supernatants were resuspended in Laemmli sample buffer with 100 mM DTT (7:3) and boiled for 5 min. For deconjugation assays 70 μl of supernatants were incubated for 2 hr at 28°C with 70 μl of *Tb*SENP supernatant with or without 25 mM N-ethylmaleimide (NEM). Finally samples were resuspended in Laemmli sample buffer with 100 mM DTT (7:3) and boiled for 5 min. Samples were analyzed by Western blot.

### Purification of SUMOylated *Sc*PCNA

Large-scale preparations were performed using 200 ml of induced cultures. To enrich for SUMOylated *Sc*PCNA, cleared lysates were loaded onto a 1 ml Ni^+2^-resin (GE Healthcare), washed with 50 column volumes (CV) of 50 mM Tris-HCl pH 7.6, 150 mM NaCl, 0.1% Tx-100, 30 mM imidazole and bound proteins were eluted by the addition of 4 CV of the same buffer containing 500 mM imidazole.

### Electrophoresis and immunoblotting

Proteins were separated by SDS-PAGE (7.5 or 10% acrylamide) followed by Coomassie Blue staining or transferred to a nitrocellulose Hybond ECL membrane (GE Healthcare, Pittsburgh, PA) for probing with high-affinity rat monoclonal anti-HA antibodies (Roche, Basel, Switzerland) diluted 1: 500, anti-Flag M2 mouse monoclonal antibody (Sigma, Saint Louis, MO, USA) diluted 1:5000, anti-polyHistidine mouse monoclonal antibody diluted 1:250 (Sigma), anti-GST mouse monoclonal antibody diluted 1:1000 (Sigma) or anti-*Tc*SUMO rabbit policlonal antibody diluted 1:500 [18]. Horseradish peroxidase-conjugated goat anti-rat, anti-rabbit or anti-mouse secondary antibody (Sigma) diluted 1:5000 was detected by chemiluminescence using SuperSignal West Pico Chemiluminescent Substrate (Pierce, Rockford, IL, USA). Prestained Protein Molecular Weight markers used were from Pierce.

### Expression and purification of recombinant proteins

The construct pBAD-*Tb*SENP-GST or pBAD-His-HA-*Tb*SUMO-GST was transformed into *E*. *coli* BL21 Codon Plus (DE3) cells. Exponential phase cultures (OD_600nm_ = 0.6) were induced with 0.2% m/v arabinose (Sigma) for 3 hr at 37°C with vigorous shaking (250 rpm). Bacteria were harvested by centrifugation and resuspended in lysis buffer (150 mM NaCl, 50 mM Tris HCl, 0.4 mg/ml lysozyme, 0.1% Triton X-100, 10 mM EDTA, 1 mM PMSF—pH 7.6) and sonicated when necessary. Samples were centrifuged at 23000 x *g* for 30 min at 4°C to obtain the bacterial crude extract. DTT was added to a final concentration of 1 mM. The recombinant *Tb*SENP-GST was purified using a glutathione-agarose resin (GE Healthcare) equilibrated with lysis buffer. The column was washed with 10 CV of TBS (50 mM Tris-HCl pH 7.6, 150 mM NaCl) and the sample was eluted with 10 mM Tris-HCl pH 8.8 containing 20 mM reduced glutathione.

The recombinant His-HA-*Tb*SUMO-GST was purified using a Ni^+2^-resin (GE Healthcare) equilibrated with lysis buffer, washed with 50 CV of 50 mM Tris-HCl pH 7.6, 150 mM NaCl, 0.1% Tx-100, 30 mM imidazole and bound proteins were eluted by the addition of 4 CV of the same buffer containing 500 mM imidazole. Eluates were loaded into a PD-10 desalting column (GE Healthcare) and then proteins were purified using a glutathione-agarose resin (GE Healthcare) equilibrated with TBS. The column was washed with 10 CV of TBS and the sample was eluted with 10 mM Tris-HCl pH 8.8 containing 20 mM reduced glutathione.

### Isopeptidase activity of *Tb*SENP

To obtain purified *Tb*SUMO conjugates to evaluate isopeptidase activity of *Tb*SENP we used PCF parasites expressing only a His-HA-tag version of *Tb*SUMO (His-HA-*Tb*SUMO cell line) [39].

About 1.2 x 10^9^ parasites were collected by centrifugation and washed once in PBS supplemented with 20 mM NEM. Cells were then resuspended in lysis buffer (6 M Urea, 500 mM NaCl, 50 mM Tris HCl, 5 mM β mercaptoethanol—pH 7.5) at a concentration of ~ 3 x 10^6^ parasites/μl and sonicated up to loss of viscosity. For further purification of *Tb*SUMO conjugates lysates were cleared by centrifugation for 30 min at 23000 x *g*. Supernatants, with the addition of imidazole at a final concentration of 20 mM, were incubated with Ni^2+^-Sepharose resin (GE Healthcare), pre-equilibrated in lysis buffer, with stirring at room temperature for 1.5 hrs. After centrifugation the resin was washed to reduce Urea concentration with buffer 1 (4 M Urea, 500 mM NaCl, 50 mM Tris HCl, 20 mM Imidazole—pH 7.50), buffer 2 (2 M Urea, 500 mM NaCl, 50 mM Tris HCl, 30 mM Imidazole—pH 7.50) and buffer 3 (500 mM NaCl, 50 mM Tris HCl, 30 mM Imidazole—pH 7.50). Proteins were eluted with buffer 4 (500 mM NaCl, 50 mM Tris HCl, 1 M Imidazole—pH 7.50) and stored at—80°C. The presence of *Tb*SUMO conjugates was confirmed by Western blot using anti-HA antibodies.

To assess the isopeptidase activity of *Tb*SENP, 0.75 μg of the purified recombinant protein were incubated with 3 μg of purified *Tb*SUMO conjugates for 1 hr at 37°C, in 30 μl of TBS containing 1 mM DTT with or without 20 mM iodoacetamide (IAA). The assay was evaluated by Western blot using anti-HA antibodies.

### Peptidase activity of *Tb*SENP

To assess the peptidase activity of *Tb*SENP, 0.75 μg of the purified recombinant protein was incubated with 7.5 μg of purified His-HA-*Tb*SUMO-GST for 1 hr at 37°C, in 30 μl of TBS containing 1 mM DTT with or without 20 mM NEM. The assay was evaluated by Western blot using anti-HA or anti-GST antibodies.

## Supporting Information

S1 Fig Anti-SUMO Western blot analysis of soluble cell extracts from induced cultures of *E*. *coli* transformed with only one plasmid pACYCDuet-1-*Tb*E1a-*Tb*E1b, pCDFDuet-1-*Tb*SUMO-*Tb*E2 or both pCDFDuet-1-*Tb*SUMO-*Tb*E2 and pACYCDuet-1-*Tb*E1a-*Tb*E1b. Both wild-type *Tb*SUMO and the lysine deficient variant *Tb*SUMOK9R were analyzed. (TIF)

S2 Fig In-bacteria SUMOylation of *Sc*PCNA. **(A)** Anti-Flag Western blot analysis of *Sc*PCNA performed on soluble cell extracts from induced (I) or uniduced (UI) cultures of *E*. *coli* transformed with pET28-*Sc*PCNA-3xFlag alone (lanes 1 and 2) or in the background of an incomplete (lane 3 and 4, pACYCDuet-1-*Tb*E1a-*Tb*E1b; lane 5 and 6, pCDFDuet-1-*Tb*SUMO-*Tb*E2) or a complete (lanes 7 and 8, pCDFDuet-1-*Tb*SUMO-*Tb*E2 plus pACYCDuet-1-*Tb*E1a-*Tb*E1b) SUMOylation system.
(TIF)

S3 Fig *In-bacteria* SUMOylation of *Tb*PCNA. **(A)** Anti-Flag Western blot analysis of *Tb*PCNA performed on soluble cell extracts from induced cultures of *E*. *coli* transformed with pET28-*Tb*PCNA-3xFlag alone (lane 4) or in the background of an incomplete (lane 1, pACYCDuet-1-*Tb*E1a-*Tb*E1b; lane 2, pCDFDuet-1-*Tb*SUMO-*Tb*E2) or a complete (lane 3, pCDFDuet-1-*Tb*SUMO-*Tb*E2 plus pACYCDuet-1-*Tb*E1a-*Tb*E1b) SUMOylation system.
(TIF)

S4 Fig *Tb*SENP peptidase activity. SUMO precursor cleavage by *Tb*SENP was evaluated *in vitro* using a *Tb*SUMO precursor produced in *E*. *coli* tagged at the N-terminus with His-HA and fused at the C-terminus to the GST protein. After purification on glutathione-agarose resin 7.5 μg of His-HA-*Tb*SUMO-GST protein was mixed with 0.75 μg of purified recombinant *Tb*SENP (produced as described in Materials and Methods) in 30 μl of TBS containing 1 mM DTT in the absence (lane 2) or presence (lane 3) of the general cysteine peptidase inhibitor N-ethylmaleimide 20 mM final concentration (NEM) and incubated at 37°C for 1 hr. Samples were analyzed by Western blot using anti-GST monoclonal antibodies. The substrate without the addition of the protease was run as a control (lane 1) and the amount of peptidase added to the reaction mixture was also run as a blank (lane 5).
(TIF)

S5 Fig Identification of SUMO Acceptor Lysines. Illustration of theoretical peptides resulting from digestion of SUMOylated targets when using **(A)** His-HA-*Tb*SUMO or **(B)** His-HA-*Tb*SUMO^T106K^ construct. Digestion of the latter leaves a diagnostic diGly tag with a mass of 114 Da attached to the modified Lys that can be identified by mass spectrometry. **(C)** Western blot analysis of *Sc*PCNA bacterial SUMOylation assay using His-HA-*Tb*SUMO or His-HA-*Tb*SUMO^T106K^ construct revealed identical patterns.
(TIF)
