# Supplementary figures and images for: Biosynthesis of SUMOylated Proteins in Bacteria Using the Trypanosoma brucei Enzymatic System
Source: PLoS One. 2015 Aug 10;10(8):e0134950. doi: 10.1371/journal.pone.0134950 (PMC4530879; doi:10.1371/journal.pone.0134950)

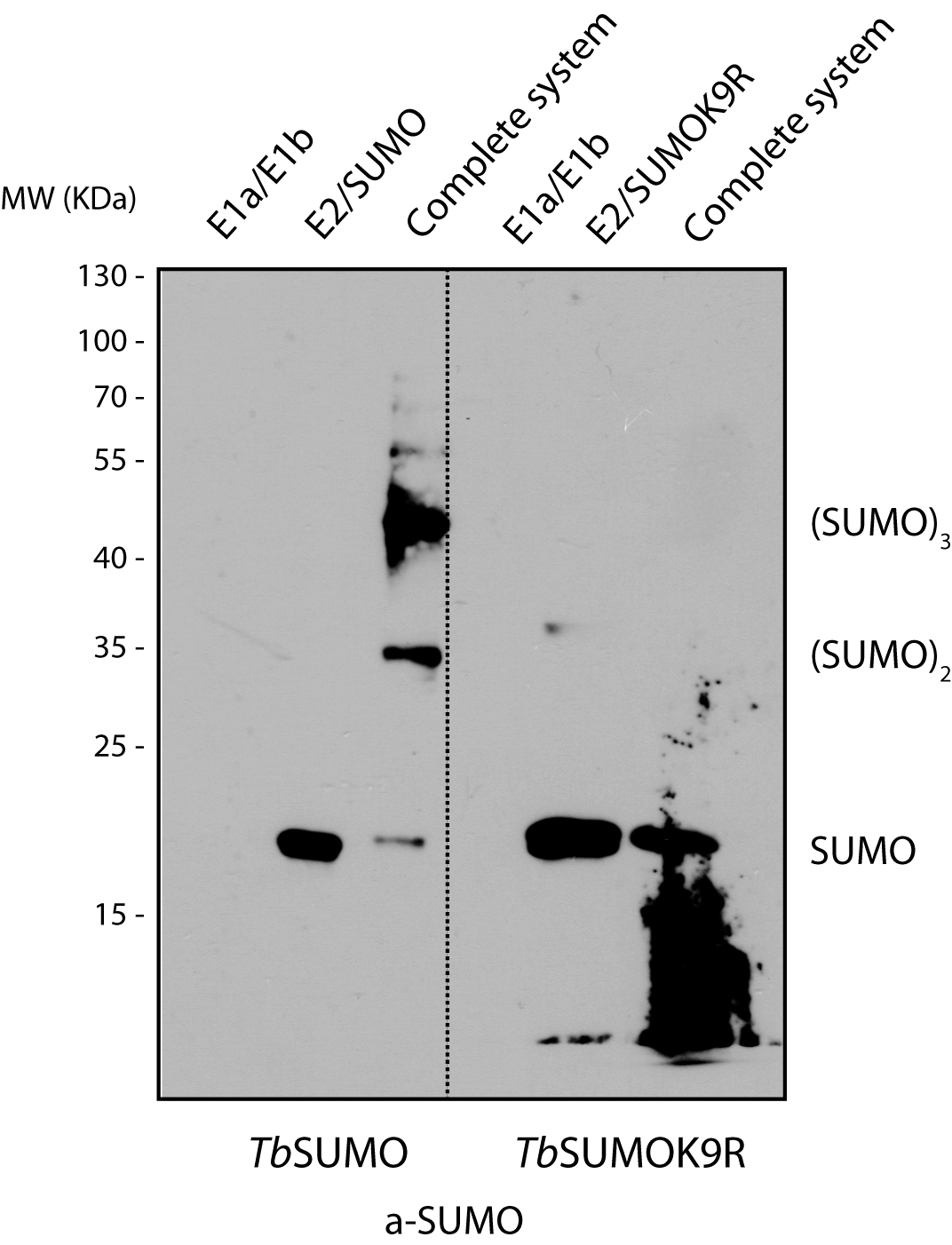

Supplement: S1 Fig — (TIF) [file pone.0134950.s001.tif]

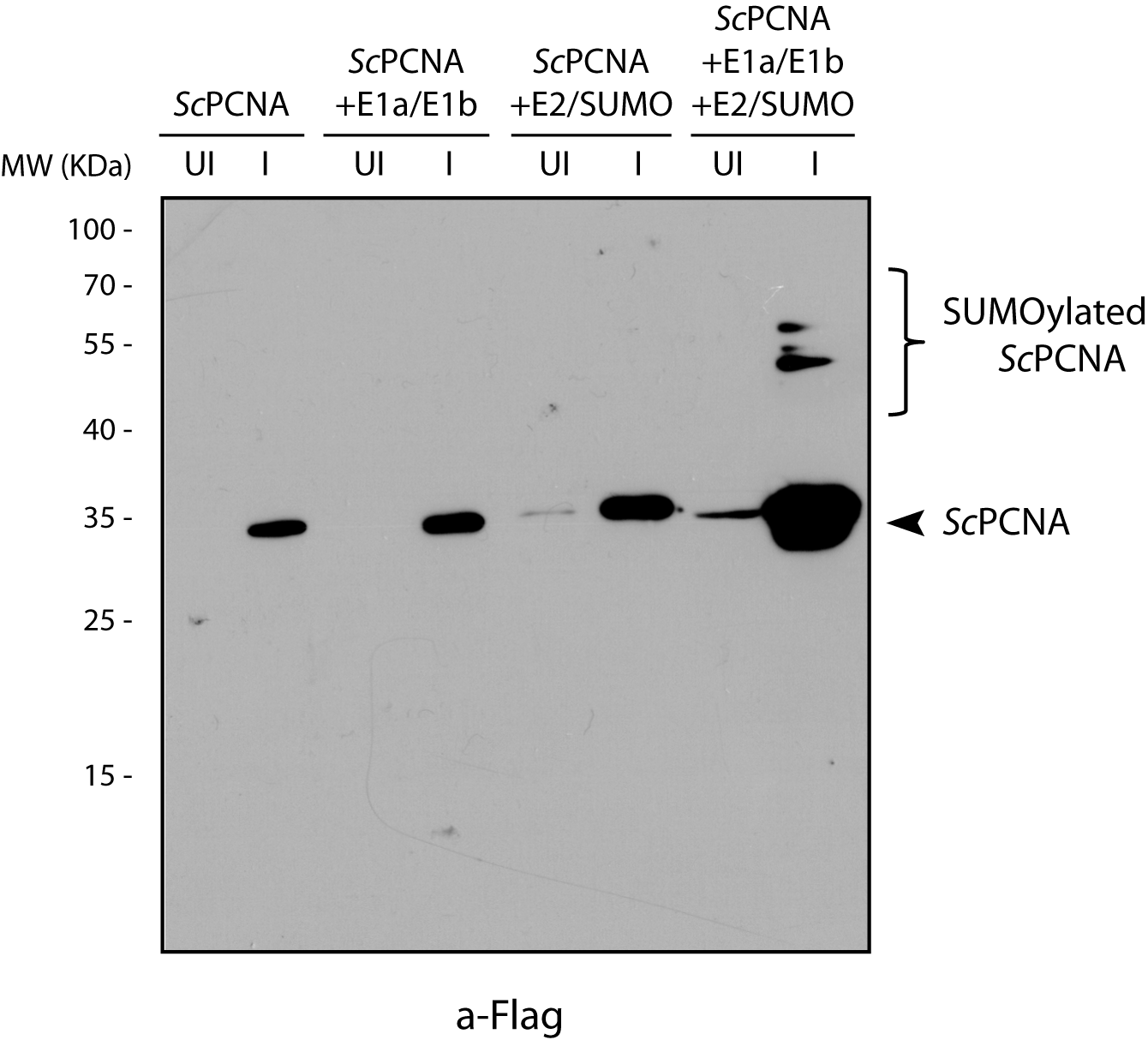

Supplement: S2 Fig — (A) Anti-Flag Western blot analysis of ScPCNA performed on soluble cell extracts from induced (I) or uniduced (UI) cultures of E. coli transformed with pET28-ScPCNA-3xFlag alone (lanes 1 and 2) or in the background of an incomplete (lane 3 and 4, pACYCDuet-1-TbE1a-TbE1b; lane 5 and 6, pCDFDuet-1-TbSUMO-TbE2) or a complete (lanes 7 and 8, pCDFDuet-1-TbSUMO-TbE2 plus pACYCDuet-1-TbE1a-TbE1b) SUMOylation system. (TIF) [file pone.0134950.s002.tif]

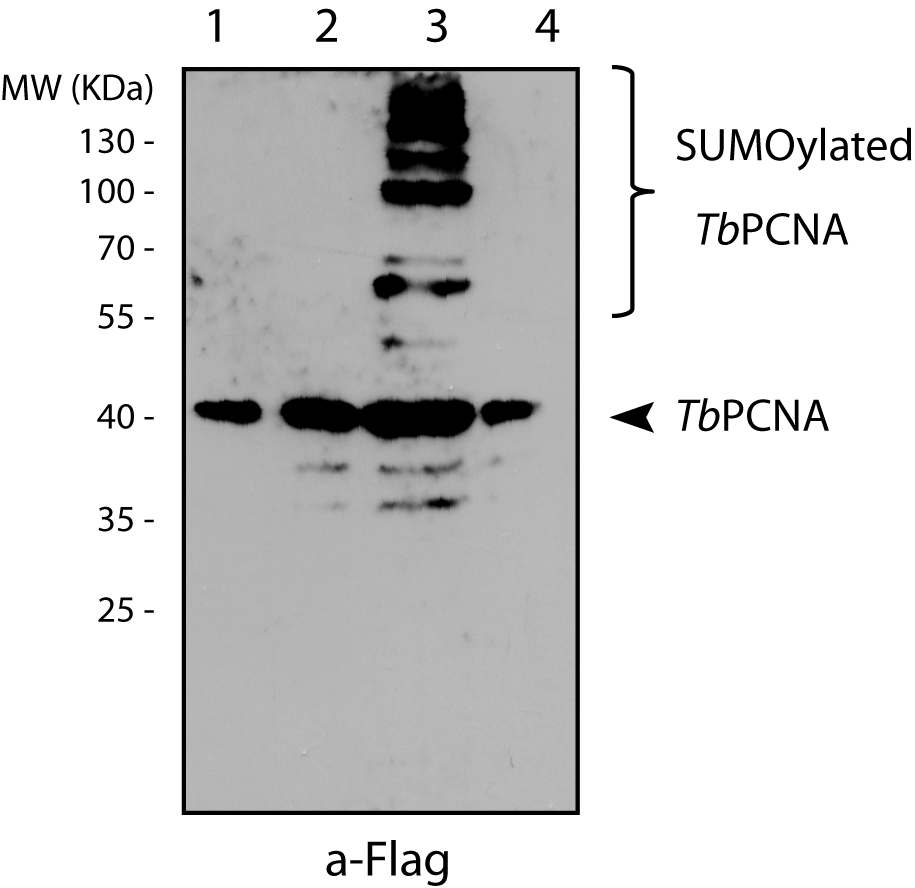

Supplement: S3 Fig — (A) Anti-Flag Western blot analysis of TbPCNA performed on soluble cell extracts from induced cultures of E. coli transformed with pET28-TbPCNA-3xFlag alone (lane 4) or in the background of an incomplete (lane 1, pACYCDuet-1-TbE1a-TbE1b; lane 2, pCDFDuet-1-TbSUMO-TbE2) or a complete (lane 3, pCDFDuet-1-TbSUMO-TbE2 plus pACYCDuet-1-TbE1a-TbE1b) SUMOylation system. (TIF) [file pone.0134950.s003.tif]

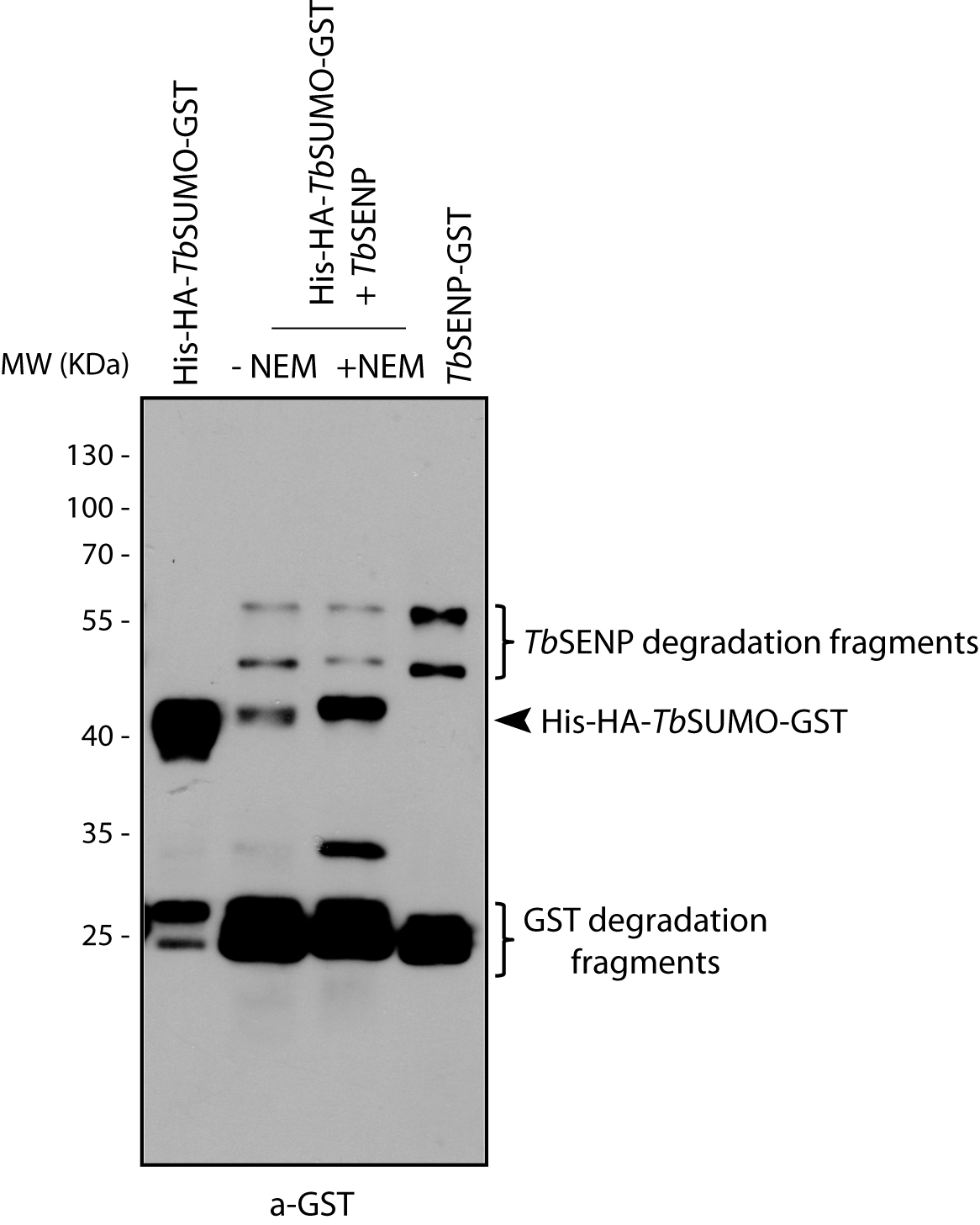

Supplement: S4 Fig — SUMO precursor cleavage by TbSENP was evaluated in vitro using a TbSUMO precursor produced in E. coli tagged at the N-terminus with His-HA and fused at the C-terminus to the GST protein. After purification on glutathione-agarose resin 7.5 μg of His-HA-TbSUMO-GST protein was mixed with 0.75 μg of purified recombinant TbSENP (produced as described in Materials and Methods) in 30 μl of TBS containing 1 mM DTT in the absence (lane 2) or presence (lane 3) of the general cysteine peptidase inhibitor N-ethylmaleimide 20 mM final concentration (NEM) and incubated at 37°C for 1 hr. Samples were analyzed by Western blot using anti-GST monoclonal antibodies. The substrate without the addition of the protease was run as a control (lane 1) and the amount of peptidase added to the reaction mixture was also run as a blank (lane 5). (TIF) [file pone.0134950.s004.tif]

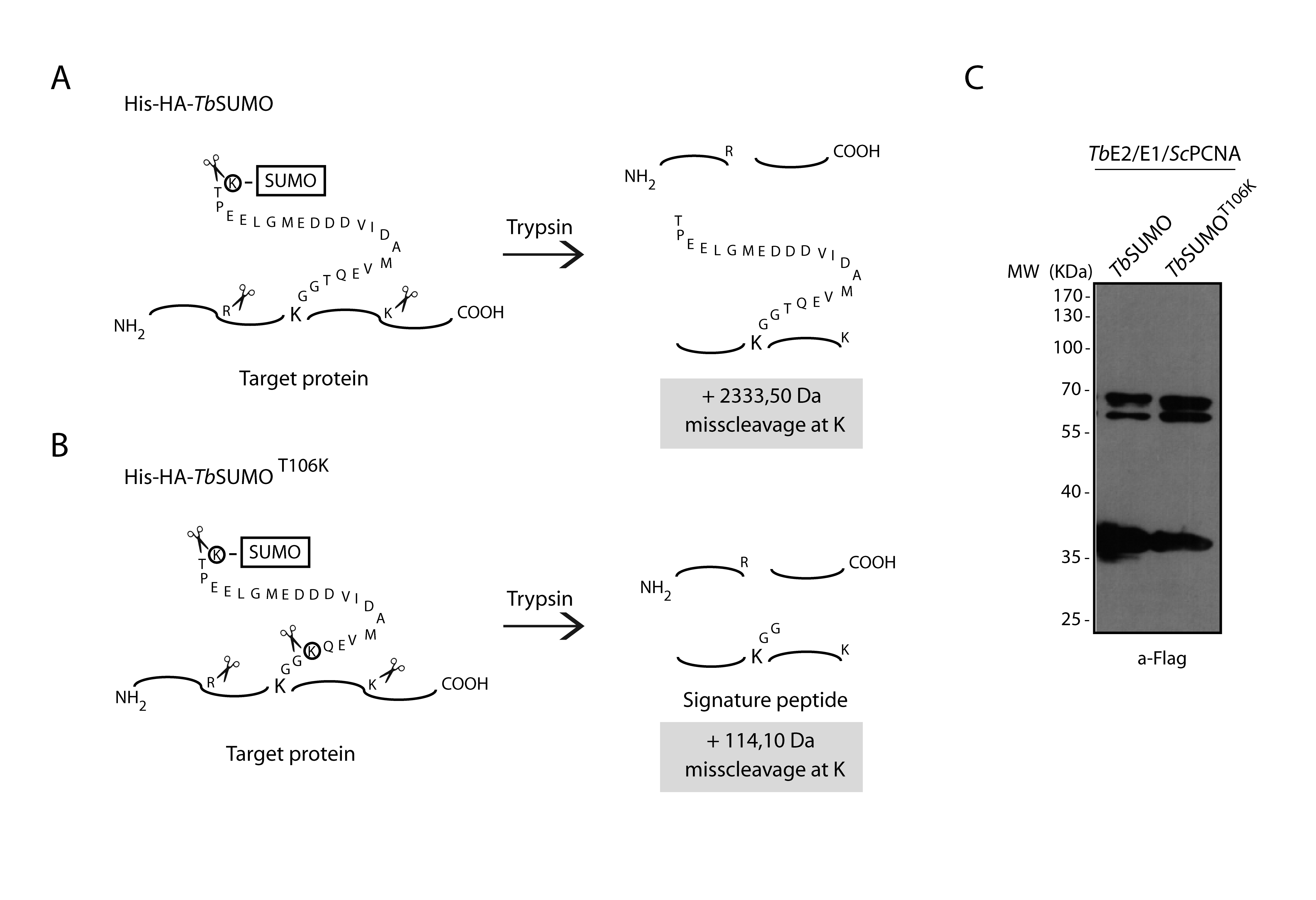

Supplement: S5 Fig — Illustration of theoretical peptides resulting from digestion of SUMOylated targets when using (A) His-HA-TbSUMO or (B) His-HA-TbSUMOT106K construct. Digestion of the latter leaves a diagnostic diGly tag with a mass of 114 Da attached to the modified Lys that can be identified by mass spectrometry. (C) Western blot analysis of ScPCNA bacterial SUMOylation assay using His-HA-TbSUMO or His-HA-TbSUMOT106K construct revealed identical patterns. (TIF) [file pone.0134950.s005.tif]
